# IGF2BP2 Drives Cell Cycle Progression in Triple‐Negative Breast Cancer by Recruiting EIF4A1 to Promote the m6A‐Modified CDK6 Translation Initiation Process

**DOI:** 10.1002/advs.202305142

**Published:** 2023-11-20

**Authors:** Tian Xia, Xin‐Yuan Dai, Ming‐Yi Sang, Xu Zhang, Feng Xu, Jing Wu, Liang Shi, Ji‐Fu Wei, Qiang Ding

**Affiliations:** ^1^ Jiangsu Breast Disease Center The First Affiliated Hospital with Nanjing Medical University 300 Guangzhou Road Nanjing 210029 China; ^2^ Department of Pharmacy Jiangsu Cancer Hospital & Jiangsu Institute of Cancer Research & The Affiliated Cancer Hospital of Nanjing Medical University Nanjing 210029 China

**Keywords:** CDK6, Cell cycle, IGF2BP2, N6‐methyladenosine (m6A)

## Abstract

IGF2BP2 is a new identified N6‐methyladenosine (m6A) reader and associated with poor prognosis in many tumors. However, its role and related mechanism in breast cancer, especially in triple‐negative breast cancer (TNBC), remains unclear. In this study, it is found that IGF2BP2 is highly expressed in TNBC due to the lower methylation level in its promoter region. Functional and mechanical studies displayed that IGF2BP2 could promote TNBC proliferation and the G1/S phase transition of the cell cycle via directly regulating CDK6 in an m6A‐dependent manner. Surprising, the regulation of protein levels of CDK6 by IGF2BP2 is related to the changes in translation rate during translation initiation, rather than mRNA stability. Moreover, EIF4A1 is found to be recruited by IGF2BP2 to promote the translation output of CDK6, providing new evidence for a regulatory role of IGF2BP2 between m6A methylation and translation. Downregulation of IGF2BP2 in TNBC cell could enhance the sensitivity to abemaciclib, a CDK4/6 inhibitor. To sum up, our study revealed IGF2BP2 could facilitate the translation output of CDK6 via recruiting EIF4A1 and also provided a potential therapeutic target for the diagnosis and treatment of TNBC, as well as a new strategy for broadening the drug indications for CDK4/6 inhibitors.

## Graphical abstract

IGF2BP2, an m6A reader, is found to be highly expressed in triple‐negative breast cancer (TNBC), which may be related to the lower methylation level in its promoter region caused by TET3. This study revealed that IGF2BP2 facilitated the translation output of CDK6 via recruiting EIF4A1 to promote TNBC proliferation, and targeting IGF2BP2 increased the sensitivity of TNBC to CDK4/6 inhibitors.

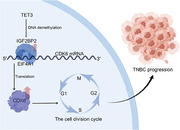

## 1 Introduction

TNBC is defined as a breast cancer with negative expression of estrogen (ER), progesterone (PR) and human epidermal growth factor receptor 2 (HER2),^[^ ^1^ ^]^ showing rapid proliferation rate, high metastatic and recurrence potential, and poor prognosis.^[^ ^2^ ^]^ The main treatment of TNBC is cytotoxic chemotherapy, while its postoperative adjuvant therapy is frequently ineffective, prone to recurrence and vulnerable to drug resistance.^[^ ^3^ ^]^ In recent years, with the use of many novel pharmaceutic preparations like poly‐ADP‐ribose polymerase (PARP) inhibitors, antibody‐drug conjugate (ADCs), immune checkpoint inhibitors (ICIs), the treatment of TNBC has been gradually enriched and exploratory.^[^ ^4^, ^5^ ^]^ Nevertheless, TNBC is still a group of molecularly genetically heterogeneous diseases. More new therapeutic options and targets are in urgent need of discovery.

M6A modification is the most common modification on mRNAs in eukaryotic cells, which plays an important role in the development of cancer.^[^ ^6^ ^]^ It is reversible and regulated by a variety of factors including m6A writers, erasers, and readers.^[^ ^7^, ^8^ ^]^ Among them, m6A readers play an important role in nuclear export, cleavage, translational export, stability and cytoplasmic localization of mRNAs by recognizing specific methylation modifications.^[^ ^8^, ^9^ ^]^ Currently identified m6A readers include the YT521‐B homology (YTH) domain family, heterogeneous nuclear ribonucleoproteins (hnRNPs), and eukaryotic initiation factor 3 (EIF3), while the insulin‐like growth factor 2‐mRNA‐binding proteins (IGF2BPs, IMPs) family is a newly discovered class of RNA‐binding proteins (RBPs) with m6A reader function.^[^ ^10^, ^12^ ^]^

​IGF2BP2 has been found to be over‐expressed in a variety of cancers and associated with their poor prognosis. For instance, Hu et al. found that IGF2BP2 could promote the proliferation of pancreatic cancer by increasing the stability of m6A‐modified differentiation antagonizing non‐protein coding RNA (DANCR) mRNA;^[^ ^13^ ^]^ Li et al. found that IGF2BP2 could synergize with the m6A writer methyltransferase like 3 (METTL3) and facilitate the progression of colorectal cancer;^[^ ^14^ ^]^ Yu et al. found that IGF2BP2 could promote lymph node metastasis in head and neck squamous carcinoma (HNSCC)^[^ ^15^ ^]^ as an m6A reader, etc. However, IGF2BP2 has only been reported as a potential biomarker associated with poor prognosis in breast cancer, and its specific mechanism of action in breast cancer, especially TNBC, has not been carefully investigated.^[^ ^15^, ^16^ ^]^

IGF2BPs are a class of RBPs including IGF2BP1/2/3 with a unique two RNA recognition motif (RRM)s and four K homology (KH) structural domains.^[^ ^17^ ^]^ Previous studies have mainly limited the regulatory mechanism of IGF2BP2 as an m6A reader to its ability to promote mRNA stability. Huang et al. initially verified that the IGF2BP family could play an active role in the translation initiation phase of targeted genes through a series of translation omics experiments.^[^ ^18^ ^]^ However, there lack clear evidences on how the IGF2BP family specifically influences and regulates this process. Wang et al. noted that translation regulation mediated by the m6A reader not only played a role in mRNAs stability, but also regulated the translation efficiency of mRNAs at the translation initiation stage to influence protein abundance. They also inferred that the YTH family may rely on the EIF4G‐mediated loop structure and interact with EIF3 to promote translation, highlighting an interesting correlation between translation initiation factors and m6A readers.^[^ ^19^ ^]^ Therefore, we speculate that IGF2BP2 has the potential to promote translation output presumably by interacting with the translation initiation factors, while the specific translation initiation factor with which it is regulated and the specific mode of action need to be investigated in detail.

In the present study, we found that IGF2BP2 was highly expressed in TNBC tissues and cell lines due to the lower methylation level in its promoter region. Functional and mechanical studies displayed that IGF2BP2 could promote TNBC proliferation and the G1/S phase transition of the cell cycle via directly regulating cyclin dependent kinase (CDK) 6 translation in an m6A‐dependent manner, indicating that the IGF2BP2‐m6A‐CDK6 axis could be a potential therapeutic target for TNBC.

## 2 Results

### 2.1 IGF2BP2 Expression was Significantly Upregulated in TNBC

**Figure 1:**
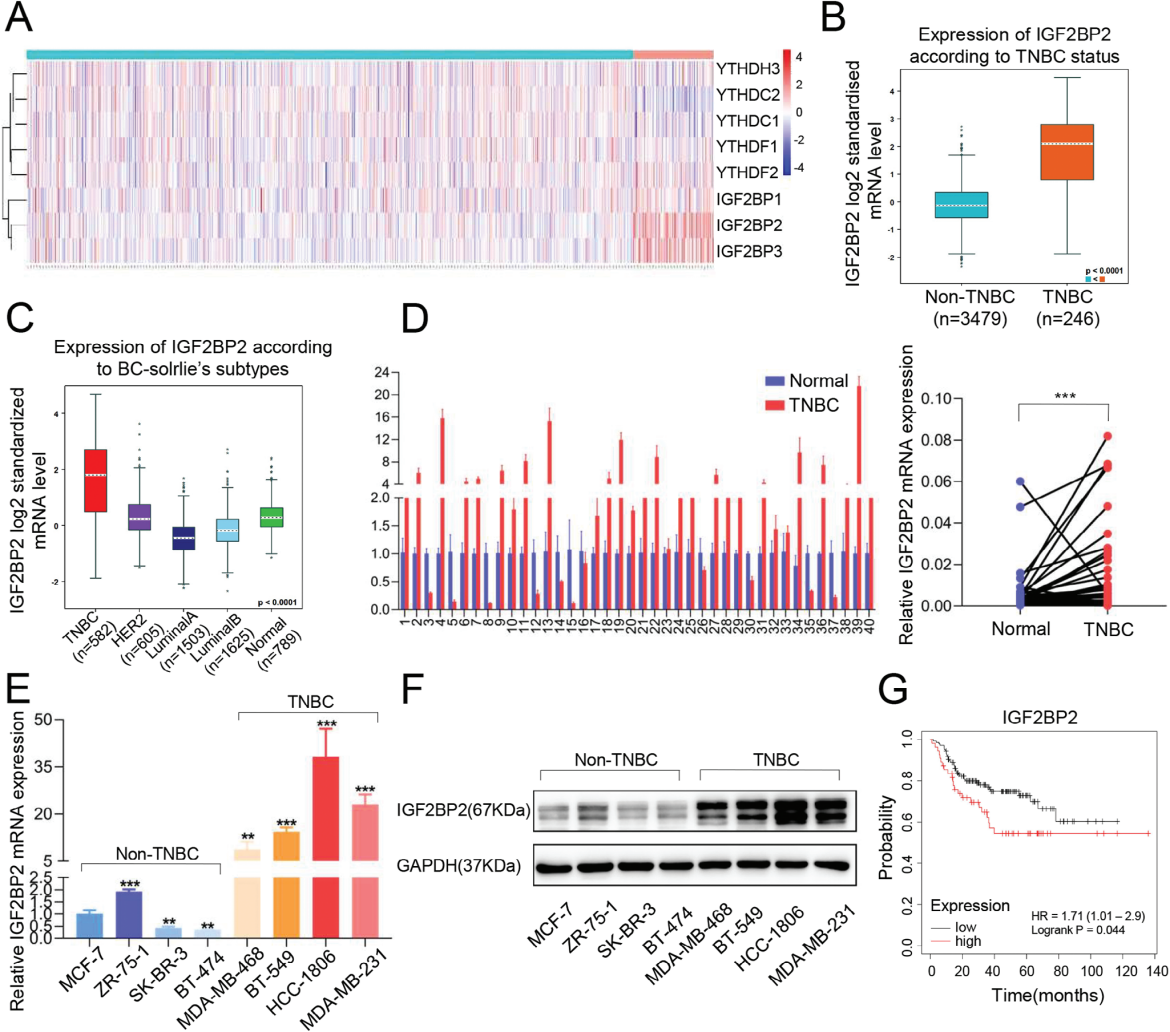
*IGF2BP2 was up‐regulated in TNBC*. (A) Heatmap of main m6A readers in TNBC and non‐TNBC based on TCGA database. (B and C) Expression of IGF2BP2 in TNBC and non‐TNBC (B), and in solrile's subtypes of breast cancer (C) analyzed by bc‐GenExMine tool. (D) ​Expression of IGF2BP2 was detected by qRT‐PCR in 40 pairs of TNBC and adjacent normal tissues. (E) qRT‐PCR analysis of IGF2BP2 mRNA expression in TNBC cell lines and non‐TNBC cell lines. The relative quantification was calculated by the 2^−ΔΔCt^ method and normalized based on β‐actin. (F) Western blot analysis of IGF2BP2 expression in TNBC cell lines and non‐TNBC cell lines. (G) Kaplan‐Meier survival analysis was used to investigate the correlation between IGF2BP2 expression and the prognosis of TNBC patients. We removed the number of patients sampled without systemic therapy and plotted the survival curves after considering the upper quartile as the low expression group (n = 145) and the rest as the high expression group (n = 55). ​High expression of IGF2BP2 was associated with a poor prognosis in TNBC, making it more difficult for patients to benefit from systemic therapy. Data were shown as mean ± SD. *p < 0.05, **p < 0.01, ***p < 0.001.

We firstly analyzed the expression of 8 classical m6A readers in 142 TNBC tissues and 696 non‐TNBC tissues based on TCGA database, and revealed that the expression of IGF2BP2 was significantly higher in TNBC than that in non‐TNBC (**Figure** **1**A). This result was also confirmed after the inclusion in the GSE96058 dataset and GSE81538 dataset (Figure 1B). The specific analysis of BC‐solrile's subtypes showed that IGF2BP2 was highly expressed in TNBC (Figure 1C). We next confirmed that IGF2BP2 was expressed higher in TNBC tissues than adjacent normal tissues of 40 patients (Figure 1D), and subsequently analyzed the correlation between IGF2BP2 and clinicopathologic factors in these samples. The results showed that there was a significant correlation between the level of IGF2BP2 expression and tumor size, while there was no statistically significant correlation with the number of axillary lymph node metastases, TNM stage, and histological grade (Figure S1A, Supporting Information). The mRNA expression and protein level of IGF2BP2 were higher in TNBC breast cell lines (especially in MDA‐MB‐231 and HCC‐1806) than those in non‐TNBC cell lines (Figure 1E,F). Kaplan‐Meier survival curves demonstrated that the overall survival of IGF2BP2 high‐expression systemically treated TNBC patients was significantly lower than the IGF2BP2 low‐expression patients (*P = 0.044) (Figure 1G).

### 2.2 DNA Demethylase TET3 Regulated the Expression of IGF2BP2

**Figure 2:**
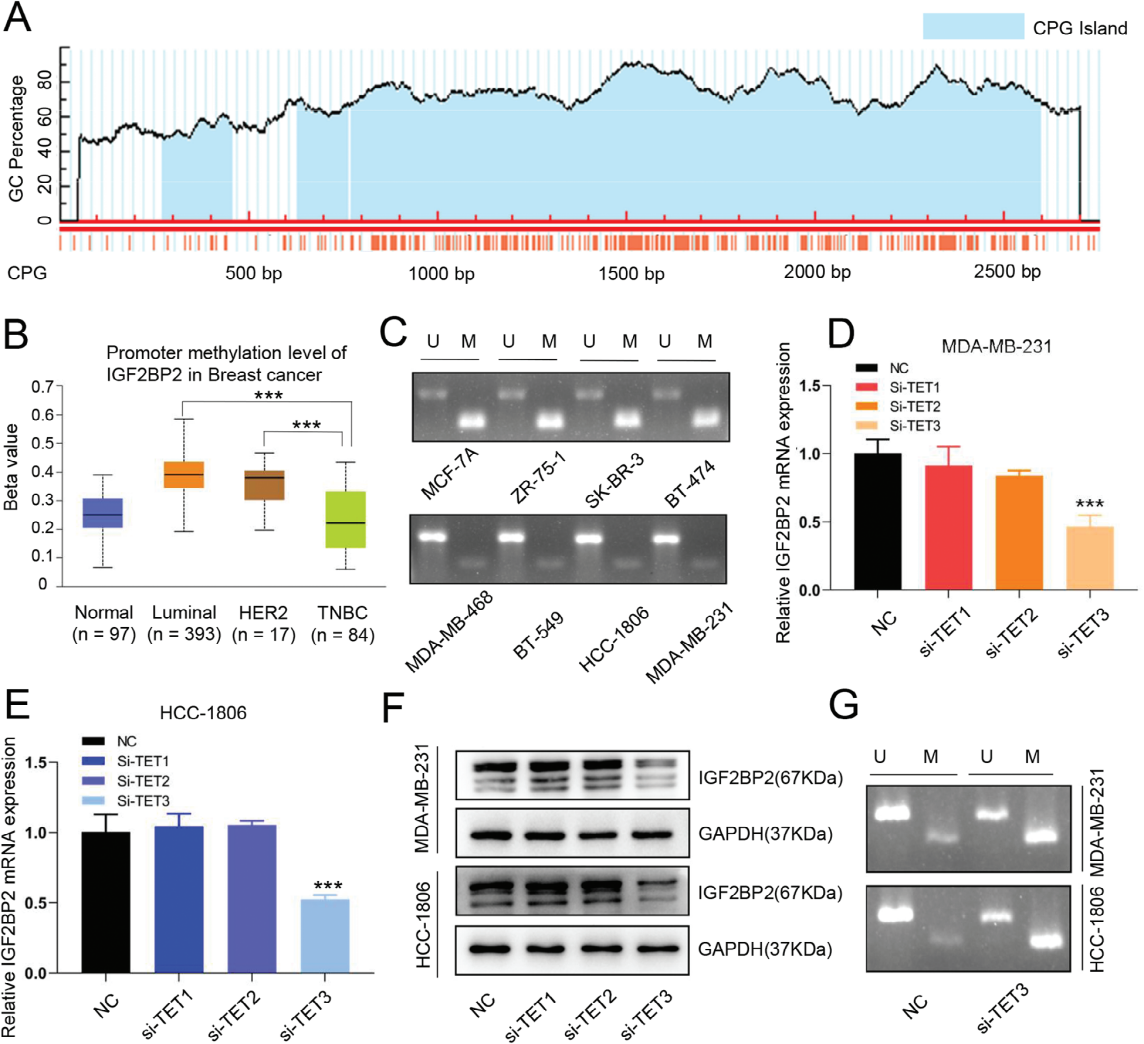
*DNA demethylase TET3 regulated the expression of IGF2BP2* (A) The distribution of CpG islands in the promoter region of IGF2BP2 gene visualized by Methprimer tool. (B) The methylation levels in the promoter region of IGF2BP2 gene in different subtypes of breast cancer based on TCGA database. (C) Agarose gel electrophoresis was used to show the methylation level of IGF2BP2 in the promoter region of non‐TNBC and TNBC cell lines. U represented unmethylation and M represented methylation. (D and E) qRT‐PCR analysis of IGF2BP2 mRNA expression in MDA‐MB‐231 (D) and HCC‐1806 cells (E) transfected with si‐TETs. (F) Western blot analysis of protein expression of IGF2BP2 transfected with si‐TETs. (G) The methylation level of IGF2BP2 in the promoter region of siTET3 and its’ blank control detected by agarose gel electrophoresis. Data were shown as mean ± SD. *p < 0.05, **p < 0.01, ***p < 0.001.

A rich distribution of CpG islands was found in the IGF2BP2 promoter region (**Figure** **2**A), which implied that IGF2BP2 expression might be regulated in DNA methylation levels. We further analyzed the methylation status of IGF2BP2 promoter region in different breast cancer subtypes, and found that the methylation level of IGF2BP2 promoter region in TNBC was lower compared with that in other molecular subtypes (Figure 2B). Then we amplified the IGF2BP2 promoter methylated and unmethylated fragments in 8 breast cancer cell lines by PCR and detected by agarose gel electrophoresis. The results showed that the IGF2BP2 promoter methylation level was significantly lower in TNBC cell lines than in non‐TNBC cell lines (Figure 2C).

Considering the DNA demethylation is closely related to the TET family of demethylases and the DNMT family of methylases, small interfering RNA (siRNA)s (siTET1, siTET2, siTET3, siDNMT1, siDNMT3A, siDNMT3B) was transfected into targeted MDA‐MB‐231 and HCC‐1806 cells. The mRNA expression (Figure 2D,E; Figure S1B, Supporting Information) and protein level (Figure 2F; Figure S1C, Supporting Information) of IGF2BP2 in these transfected cells was significantly downregulated in cells transfected with siTET3. Western blot further confirmed the transfection efficiency of siTET1/2/3 (Figure S1D, Supporting Information). Further detecting the methylation level of IGF2BP2 promoter of siTET3 and its’ blank control in MDA‐MB‐231 and HCC‐1806 cell lines by agarose gel electrophoresis, and it was found that the methylation level was significantly increased after TET3 knockdown (Figure 2G). Interestingly, TET3 was also highly expressed in TNBC based on BC‐solrile's subtypes (Figure S1E, Supporting Information). These results suggested that TET3 upregulated IGF2BP2 expression by reducing the methylation level of its promoter region in TNBC cells, which also provided a possible explanation for the high expression of IGF2BP2 in TNBC.

### 2.3 IGF2BP2 could Promote TNBC Cells Proliferation and G1/S Transition of Cell Cycle Progression

**Figure 3:**
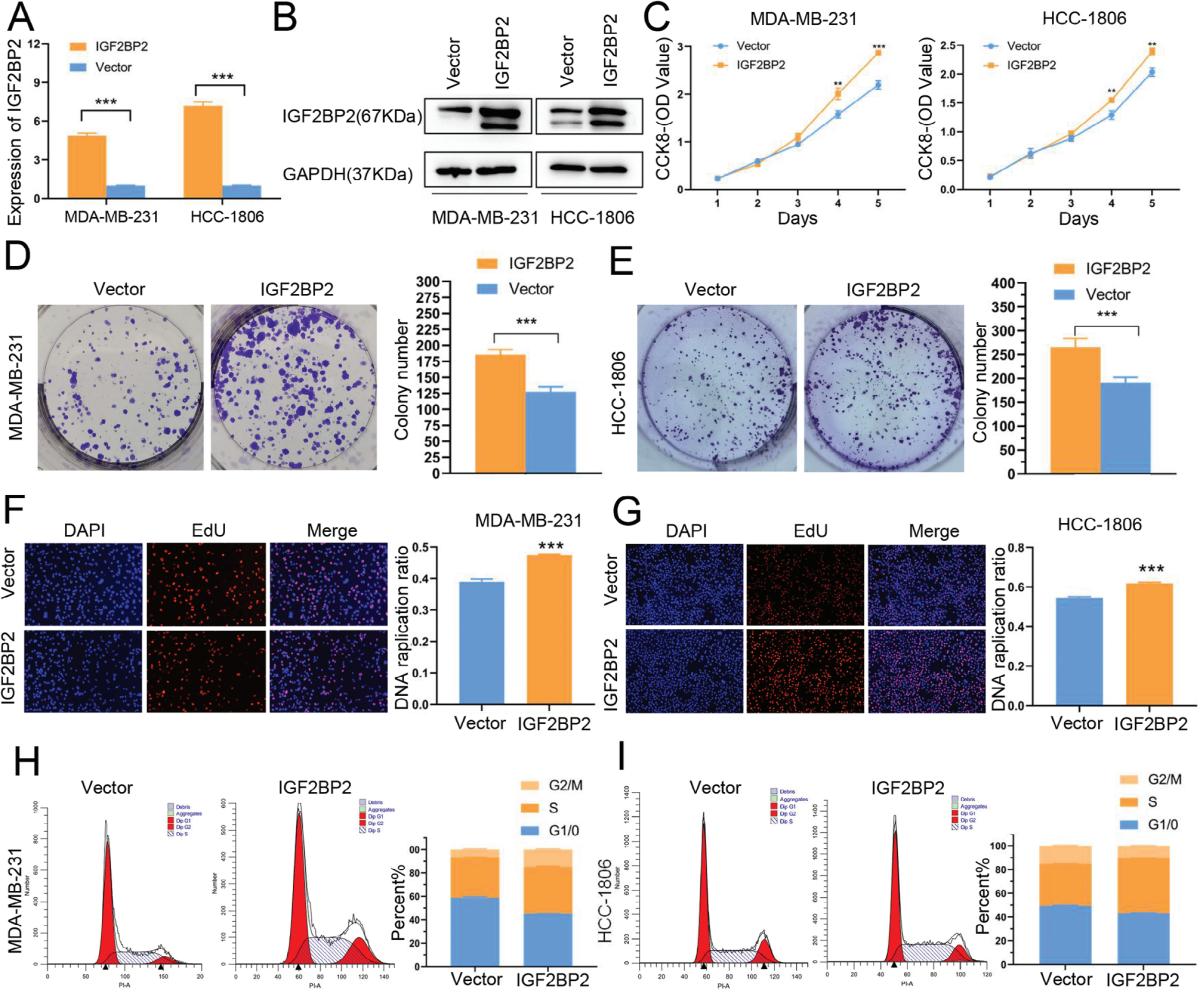
*Overexpression of IGF2BP2 could premote TNBC cells proliferation* (A and B) The expression of IGF2BP2 in MDA‐MB‐231 and HCC‐1806 cells transfected with IGF2BP2 overexpression (IGF2BP2) and blank control (Vector) were detected by qRT‐PCR (A) and Western blot (B). (C) CCK8 assays were used to detect the cell viability after overexpression of IGF2BP2 over 5 days. (D and E) Representative colony formation results showed the proliferation of MDA‐MB‐231 (D) and HCC‐1806 (E) cells after overexpression of IGF2BP2. (F and G) EdU assays were performed to identify the growth rates of MDA‐MB‐231 (F) and HCC‐1806 (G) cells transfected with IGF2BP2 overexpression. (H and I) Flow cytometry analysis of cell cycle in MDA‐MB‐231 (H) and HCC‐1806 (I) cell lines transfected with IGF2BP2 overexpression. Data were shown as mean ± SD. *p < 0.05, **p < 0.01, ***p < 0.001.

**Figure 4:**
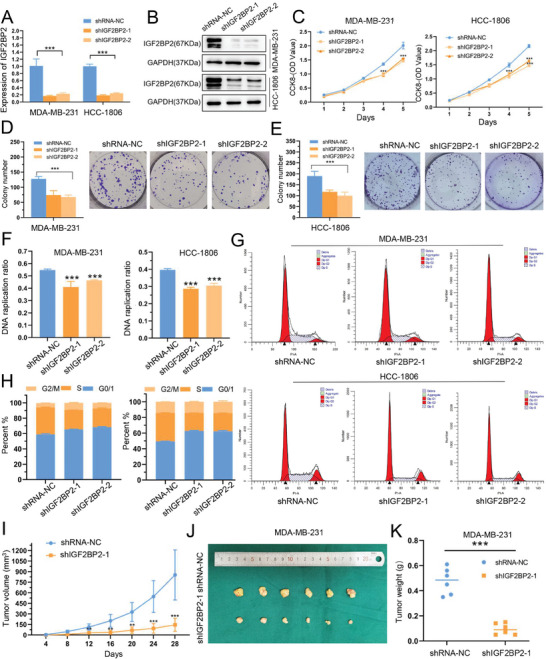
*Knockdown of IGF2BP2 could inhibit TNBC cells proliferation* in vitro *and* in vivo (A and B) The levels of IGF2BP2 in MDA‐MB‐231 and HCC‐1806 cells transfected with IGF2BP2 knockdown (shIGF2BP2‐1, shIGF2BP2‐2) and negative control (shRNA‐NC) were detected by qRT‐PCR (A) and Western blot (B). (C) The growth of MDA‐MB‐231 and HCC‐1806 cells after knockdown of IGFB2P2 over 5 days was measured using CCK8 assays. (D and E) The growth of cells over 14 days after knockdown of IGF2BP2 were measured using colony formation assays. (F) Knockdown of IGF2BP2 inhibited DNA replication. (G‐H) Knockdown of IGF2BP2 led to cell cycle arrest in both MDA‐MB‐231 and HCC‐1806. Cell cycle was measured by flowcytometry. (I) Subcutaneous tumor growth in nude mice was recorded every 4 days. (J‐K) The excised tumor lumps were photographed (J) and weighed (K). Data were shown as mean ± SD. *p < 0.05, **p < 0.01, ***p < 0.001.

To investigate the role of IGF2BP2 in TNBC proliferation, IGF2BP2 overexpression or knockdown lentivirus were transfected into MDA‐MB‐231 and HCC‐1806 cells. QRT‐PCR (**Figure** **3**A and **Figure** **4**A) and western blot (Figure 3B and Figure 4B) were used to verify the transfection efficiency. According to the growth curves derived from the CCK‐8 assay, IGF2BP2 overexpression promoted the proliferation of MDA‐MB‐231 and HCC‐1806 cell lines, while knockdown of IGF2BP2 could slow down the growth rate of these two cell lines (Figure 3C and Figure 4C). In addition, knockdown of IGF2BP2 impaired the colony‐forming ability of both cell lines, whereas overexpression of IGF2BP2 enhanced this ability (Figure 3D,E, Figure 4D,E). Similar results were obtained in the EdU assays, where the DNA replication rate was downregulated in the knockdown cell line and increased in the overexpression cell line (Figure 3F,G, Figure 4F; Figure S2A, Supporting Information). Next, we used flow cytometry to identify if IGF2BP2 could affect the progression of cell cycle. The results showed that significant downregulation of IGF2BP2 increased the number of cells in G0/G1 phase, while the number of cells in S and G2 phases increased after overexpression of IGF2BP2 (Figure 3H,I, Figure 4G,H). Furthermore, we examined the expression of topic cell cycle proteins [CDK4, CDK6, CCND1, phospho‐retinoblastoma (pRb)] in the G1/S phase progression and found upregulation of CDK6 and pRb after overexpression of IGF2BP2 (Figure S2B,C, Supporting Information). We inferred that IGF2BP2 could drive the cell transition from G1/S phase and thus promoted cell proliferation. Finally, we constructed xenograft tumor transplantation models of the knockdown cell line and its control group in nude mice. The IGF2BP2 knockdown group had significantly slower tumor growth than the control group (Figure 4I). Its tumor size and mass were also significantly lower in IGF2BP2 knockdown group (Fig. 4J and K). Additionally, the levels of IGF2BP2 in different groups of tumors were measured by western blot to ensure the validity of the in vivo experiments (Figure S2D, Supporting Information).

### 2.4 CDK6 was Identified as a Direct Target of IGF2BP2 in TNBC Cells

**Figure 5:**
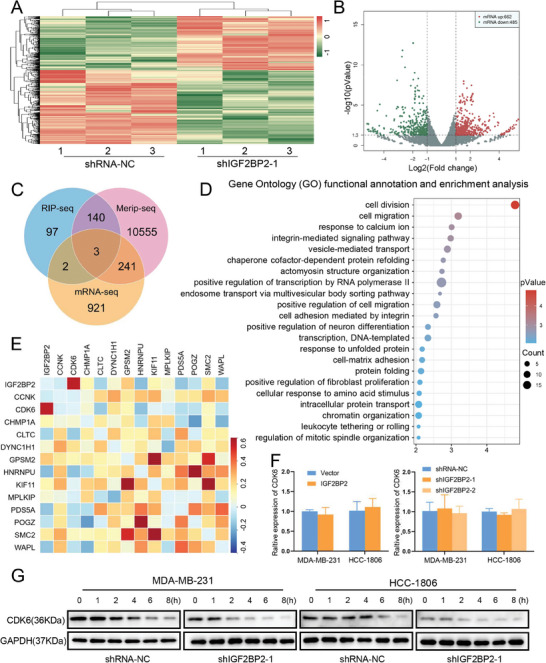
*Multiomic analyses identified IGF2BP2 targets involved in TNBC*. (A and B) The results of mRNA‐seq were presented in the form of heat map (A) and volcano map (B), where red represents upregulated gene expression and green represents downregulated gene expression. (C) Venn diagram illustrating the overlap between differentially expressed genes by mRNA‐seq, genes containing m6A peaks by MeRIP‐seq, and IGF2BP2‐bound genes by IGF2BP2 RIP‐seq. (D) The DAVID website (https://david.ncifcrf.gov) was used for clustering analysis of potential targets of IGF2BP2 and Bubble plots showed the results of GO enrichment analysis. (E) Correlation matrix displayed the correlation of 13 possible target genes with IGF2BP2 in breast cancer. (F) The mRNA expression of CDK6 in MDA‐MB‐231 and HCC‐1806 cell lines transfected with IGF2BP2 overexpression and knockdown detected by qRT‐PCR. (G) Using Cycloheximide (CHX) to test the stability of CDK6 protein in shGF2BP2 transfected MDA‐MB‐231 and HCC‐1806 cells, results were showed by western blot.

To further clarify the specific role of IGF2BP2 plays in m6A regulatory modifications, we performed MeRIP‐seq and RIP‐seq using the MDA‐MB‐231 cell. In addition, mRNA‐seq of the IGF2BP2 knockdown group and control group in MDA‐MB‐231 cells were used to search potential mRNA targets mediated by IGF2BP2 (**Figure** **5**A,B). Cross‐combination analysis of mRNA‐seq/MeRIP‐seq/RIP‐seq showed that 143 genes were labeled by m6a‐modification, while RNA abundance was altered by IGF2BP2 in only 3 genes (Figure 5C). We performed Gene Ontology (GO) functional annotation and enrichment analysis using DAVID on 140 genes with unaffected RNA abundance, and presented them in bubble plots (Figure 5D). It could be found that the p‐value of cell division (GO: 0051301) ranked first, and the detailed list of 13 genes was put in the following figure (Figure S3A, Supporting Information).

As seen in the correlation matrix of IGF2BP2 with 13 genes in TNBC, CDK6 ranked first (Figure 5E). While CDK6 played a key role in the G1/S phase transition of the cell cycle, which consistent with our finding that IGF2BP2 could promote the cell cycle progress. Further exploration of the CPTAC database discovered that the protein levels of CDK6 were remarkably higher in TNBC than in normal tissue and other subtypes of breast cancer (Figure S3B, Supporting Information). The mRNA expression (Figure 5F) and protein level (Figure S2B,C, Supporting Information) of CDK6 in the IGF2BP2 lentiviral transfected cell lines of MDA‐MB‐231 and HCC‐1806 showed a positive correlation between IGF2BP2 and CDK6 at the protein level, but not on the transcriptional level. To rule out that IGF2BP2 might affect the protein stability of CDK6, we used cycloheximide to block the translation of MDA‐MB‐231 and HCC‐1806 cell lines and assayed the quantity of CDK6 protein in shRNA‐NC and shIGF2BP2‐1. The results showed that CDK6 decreased at a similar rate in control and knockdown groups (Figure 5G; Figure S3C, Supporting Information). This result demonstrated that knockdown of IGF2BP2 led to a decrease in CDK6 protein levels not due to the effect on the output of the protein itself but rather on the translational levels.

### 2.5 IGF2BP2 Promoted CDK6 mRNA Translation in an m6A‐Dependent Manner

**Figure 6:**
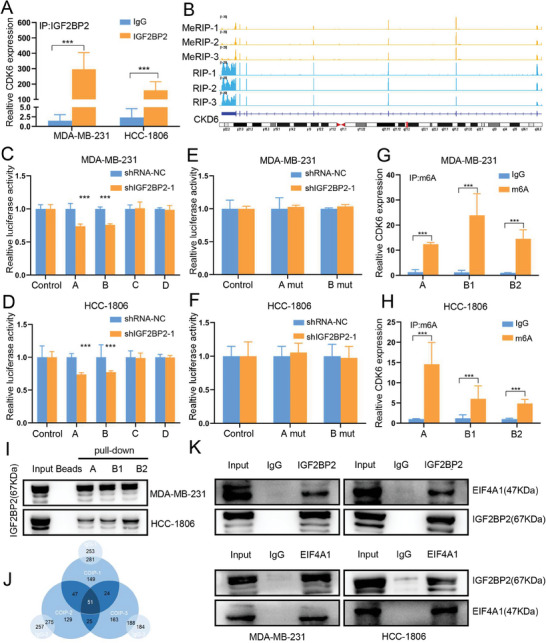
IGF2BP2 Promoted *CDK6 Translation in an m6A‐Dependent Manner*. (A) qRT‐PCR measured transcript levels of CDK6 within IGF2BP2 or IgG immunocomplexes in MDA‐MB‐231 and HCC‐1806 cell lysates. The relative levels of all genes are normalized with IgG. (B) Distribution of m6A peaks and IGF2BP2‐binding peaks across CDK6 transcript, results were showed by Integrative Genomics Viewer (IGV). (C and D) The reporters containing the possible m6A binding sites between IGF2BP2 and CDK6 mRNA was decreased after knockdown of IGF2BP2 in MDA‐MB2‐231(C) and HCC‐1806 (D) cell lines. Firefly luciferase activity was detected and normalized to renilla luciferase activity. A was located in the 5′‐UTR, B and C in the CDS region, and D in the 3′‐UTR. (E and F) The key sequences of the m6A modification site were mutated and detected again, which finally confirmed that A and B were the m6A binding sites of CDK6 mRNA recognized by IGF2BP2. (G and H) MeRIP‐qRT‐PCR and semiquantitative PCR analysis of m6A site in A and B region on CDK6 mRNA. (I) RNA pull‐down with a biotin‐labeled CDK6 probes followed by western blot to test the enrichment of IGF2BP2. Both probes A and B were designed based on the binding sites described above, in which B sequence was split into B1 and B2 probes because of its excessive length. (J) IP‐MS was used to identify the possible interacting proteins of IGF2BP2, results were presented in the form of Venn diagrams. (K) Co‐immunoprecipitation (Co‐IP) and western blot had shown the binding of IGF2BP2 and CDK6 in MDA‐MB‐231 and HCC‐1806 cell lines. Data were shown as mean ± SD. *p < 0.05, **p < 0.01, ***p < 0.001.

To further determine the binding of IGF2BP2 protein with CDK6 mRNA, RIP assays was used to detected the mRNA enriched by IGF2BP2, and the results were illustrated using qRT‐PCR and agarose gel electrophoresis (Figure 6A; Figure S4A, Supporting Information). Through the detailed analysis of m6A modification of CDK6 in MeRIP‐seq and RIP‐seq, and the predictive analysis of potential binding targets based on SCRAMP tool, four m6A modification sites were ultimately determined (Figure 6B; Figure S4B, Supporting Information). We cloned the above predicted position sequence into the Pgl3 vector with the firefly luciferase reporter gene vector and performed Dual‐luciferase reporter assay on MDA‐MB‐231 and HCC‐1806 shRNA‐NC and shIGF2BP2‐1 cell lines (Figure 6C,D). We then mutated the key m6A modification sites in A and B sequences and reconstructed the firefly luciferase reporter gene vectors for transfection. The results showed that the luciferase activity of the reporters containing mutant sequences were similar between the IGF2BP2 knockdown group and control group (Figure 6E,F). MeRIP assay and qRT‐PCR verified that the key recognition sequences of the A and B sequences screened in the above experiments had indeed m6A modifications (Figure 6G,H). In addition, three biotinylated RNA probes were designed (Figure S4C, Supporting Information) based on the sequences of A and B, and RNA‐pulldown assays was performed in MDA‐MB‐231 and HCC‐1806 wild type cells. It showed that the m6A‐modified fragment on CDK6 mRNA could bind to IGF2BP2 through western blot (Figure 6I).

Translation initiation is the most important rate‐limiting step in the translation process. The translation initiation located in the 5′‐untranslated regions (UTR) is rather dependent on the cap structure. The 5′‐UTR of CDK6 has long and complex secondary structure, especially the presence of many G‐quadruplex structures (Figure S4D,E, Supporting Information). We thus hypothesized that IGF2BP2 could functionally interact with the cap structure‐associated proteins to enhance the translation efficiency of CDK6 mRNA. Fifty‐one proteins were identified by immunoprecipitation tandem mass spectrometry (IP‐MS) that interact with IGF2BP2 protein (Figure 6J), and finally identified EIF4A1, an important core functional protein in the cap structure‐dependent translation initiation factor complex. The results of western blot further confirmed that IGF2BP2 protein interacted with EIF4A1 protein (Figure 6K).

The above results conclusively established that IGF2BP2 was able to recognize and bind to m6A modification sites in the 5′ UTR and protein‐coding sequence (CDS) region on CDK6 mRNA, and recruited the translation initiation factor EIF4A1 protein to unwind its complex 5′ UTR region structure to facilitate its translation.

### 2.6 Upregulation of CDK6 Reversed the Proliferation Inhibition of IGF2BP2‐Knockdown TNBC Cells

**Figure 7:**
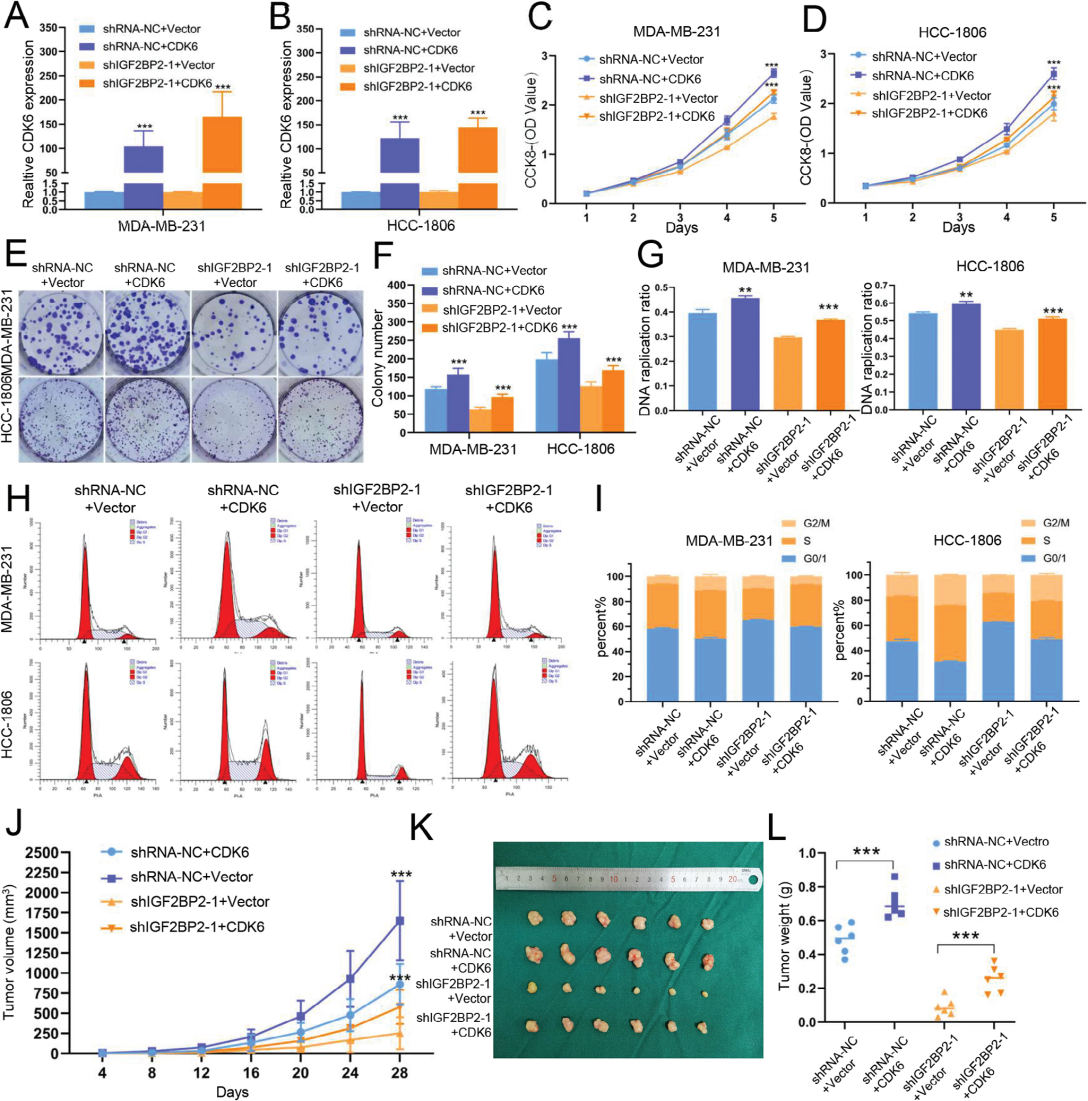
*Upregulated of CDK6 reversed the proliferation inhibition of IGF2BP2‐knochdown TNBC cells*. (A and B) qRT‐PCR were used to verify the efficiency of CDK6 overexpression. (C and D) Cell proliferation assay in knockdown of IGF2BP2 or control MDA‐MB‐231 (C) and HCC‐1806 (D) cells after transfection with CDK6 by CCK‐8 assays. (E and F) Colony formation assays in knockdown of IGF2BP2 or control cells after transfection with CDK6. Representative photographs (E) and quantification (F) were shown. (G) Overexpression of CDK6 in shIGF2BP2‐1 rescued inhibited DNA replication. (H and I) Overexpression of CDK6 in shIGF2BP2‐1 rescued cell cycle arrest. Representative photographs (H) and quantification (I) were shown. (J‐L) Tumor volumes were measured in different treatment groups (J), and the excised tumor lumps were weighed (K) and photographed (L) with the overexpression of CDK6 in shIGF2BP2‐1. Data were shown as mean ± SD. *p < 0.05, **p < 0.01, ***p < 0.001.

To further investigate whether the role of IGF2BP2 in promoting TNBC proliferation was indeed mediated by CDK6, we designed CDK6 overexpression plasmids and transfected knockdown cell lines and their controls, which were assayed at the mRNA expression and protein levels (Figure 7A,B; Figure S5A,B, Supporting Information). The colony formation assays, CCK‐8 assays and EdU assays indicated that upregulation of CDK6 reversed the inhibition of proliferation by knocking down IGF2BP2 (Figure 7C–G; Figure S5C,D, Supporting Information). We also detected cell cycle changes by flow cytometry and showed that overexpression of CDK6 reduced the proportion of G1/0 phase and increased G2/S phase, thus reversing the G1/S phase block caused as a result of knockdown of IGF2BP2 (Figure 7H,I). This conclusion was also confirmed by the detection of the expression of topic G1/S phase‐related proteins (Figure S5A,B, Supporting Information). Lastly, we constructed nude mouse xenograft tumor models and compiled observational statistics, with the same trend observed in the in vitro model (Figure 7J–L; Figure S5E, Supporting Information).

### 2.7 IGF2BP2 could Increase the Drug Sensitivity of TNBC Cells to abemaciclib

We examined the IC50 values of abemaciclib (LY2835219) in the IGF2BP2 knockdown and control cells of MDA‐MB‐231 and HCC‐1806 cell lines, and the IC50 values in both cell lines were decreased among shIGF2BP2‐1 compared with shRNA‐NC (Figure S6A,B). Therefore, we hypothesized that the inhibition of IGF2BP2 in TNBC can sensitize TNBC cells to abemaciclib due to the reduction of CDK6 protein expression.

## 3 Discussion

Reports on the specific role of IGF2BP2 in TNBC are scarce, and the mechanism of how its role as an m6A reader facilitates the translation of targeted genes is poorly understood. In this study, we found a newly authenticated m6A reader, IGF2BP2, was highly expressed due to the lower methylation level in its promoter region. Moreover, IGF2BP2 could promote the proliferation of TNBC in vivo and in vitro and implied a poor prognosis in TNBC patients. Mechanically, CDK6 was found to be targeted by IGF2BP2 as the key role in the G1/S phase, and this regulatory role was regulated in the translation level by an m6A‐dependent manner.

IGF2BP2 has been shown to play an essential role in the progression of multiple cancers.^[^ ^20^ ^]^ Its role in the rapid proliferation of colorectal, pancreatic, thyroid, gastric, prostate, and other tumors is also well documented.^[^ ^13^, ^14^, ^21^, ^22^, ^23^ ^]^ Liu et al. found an association between IGF2BP2 and poor breast cancer prognosis based on the detection of genomic DNA from peripheral blood in Chinese women.^[^ ^24^ ^]^ Almawi et al. also confirmed the predictive role of IGF2BP2 in the poor prognosis of breast cancer from genomic DNA testing in peripheral blood, and found differential gene frequencies of IGF2BP2 in TNBC and non‐TNBC patients.^[^ ^25^ ^]^ And further analysis of the relationship between clinicopathological data and IGF2BP2 expression in our TNBC samples revealed that tumor size was significantly correlated with IGF2BP2. And the correlation between IGF2BP2 expression and lymph node metastasis, TNM stage was not statistically significant although there was a certain trend. Considering the factors of early stage and insufficient sample size of patients with triple‐negative breast cancer with indications for surgery, it is predicted that it is necessary to continue to expand the sample size in follow‐up in order to support in‐depth research. Moreover, our study identified the differences in IGF2BP2 expression in TNBC and non‐TNBC tissues and cell lines. We found that IGF2BP2 methylation levels were significantly lower in TNBC than in non‐TNBC by analyzing the methylation levels in IGF2BP2 promoter regions. We thus speculated that the abnormal expression of IGF2BP2 in TNBC might be related to the methylation levels in its promoter regions, a change that had also been experimentally confirmed to be triggered by the demethylating enzyme TET3, which was also highly expressed in TNBC. Admittedly, there must be other regulatory mechanisms affecting the expression level of IGF2BP2 in TNBC, such as those brought about by acetylation, ubiquitination modification, etc., which deserves broader studies in the future.

We identified the role of IGF2BP2 in promoting TNBC proliferation through a series of functional experiments, and characterized its specific effects on cell cycle progression. We observed a significant increase in the number of cells in G1 phase after knockdown of IGF2BP2, blocking the cell cycle during the G1 to S phase transition. Coincidentally, CDK6, which was confirmed to be the targeted gene of IGF2BP2 in our study, was one of the key cyclins in cell cycle progression. CDK6 is an essential member of the cyclin‐dependent kinase family (CDKs), like its highly homologous CDK4, activation by CCND1/2/3 binds and phosphorylates retinoblastoma (Rb) to inactivate it, thereby driving progression through the G1/S phase of the cell cycle.^[^ ^26^ ^]^ And synthesizing our experimental results demonstrated that IGF2BP2 affects the translational output process of CDK6 precisely by targeting it, thus affecting the protein expression level of pRb, which ultimately influences the progression of the G1/S phase of the cell cycle. This was consistent with the western blot results on the expression levels of topic proteins in the G1/S phase.

At present, CDK4/6 inhibitors include palbociclib (PD0332991), ribociclib (LEE011) and abemaciclib (LY2835219) had been officially approved by the FDA and commercialized for treating breast cancer.^[^ ^27^, ^28^ ^]^ Theoretically, TNBC has a higher rate of DNA replication and cycle progression in tumor cells is rather dependent on the CDK4/6‐CCND complex, which provides a conceptual basis for related targets that can be applied to TNBC.^[^ ^29^ ^]^ However, the efficacy of CDK4/6 inhibitors in TNBC is not satisfactory, and how to expand the indications of this drug in TNBC has become one of the hot topics of clinical research today.^[^ ^30^ ^]^ Clinical trials have been conducted for the treatment of LAR TNBC with CDK4/6 inhibitors in combination with finasteride.^[^ ^31^ ^]^ Zhu et al. also investigated that palbociclib could be combined with the PARP inhibitor olaparib in BRCA‐mutated TNBC and provided a strong theoretical basis for clinical application.^[^ ^32^ ^]^ Surprisingly, we found that knockdown of IGF2BP2 in TNBC cells could reduce their resistance to abemaciclib. Intriguingly, Yang et al. found that CDK6 amplification was present in abemaciclib resistant strains, and that lowering CDK6 was able to increase the susceptibility of resistant strains to abemaciclib.^[^ ^33^ ^]^ Following this study, Li et al. found that CDK6 amplified in drug‐resistant strains could trigger resistance to abemaciclib and palbocicelli in tumor cells through the induction and binding of the CDK inhibitor protein INK4.^[^ ^34^ ^]^ Therefore, we implied that the knockdown of IGF2BP2 was accompanied by a decrease in CDK6 protein levels, which presumably increased the drug sensitivity of TNBC cells to CDK4/6 inhibitors. It is undeniable that resistance mechanisms are heterogeneous in different cell lines, and in vitro experiments in which IGF2BP2 was knocked down to increase the cell sensitivity to abemaciclib were not particularly prominent. This still provided us with new ideas to use IGF2BP2 as a possible therapeutic target for expanding the application of CDK4/6 inhibitors in TNBC.

IGF2BP2 has long been investigated as RNA‐binding proteins to promote the mRNA stability of targeted genes.^[^ ^12^, ^18^, ^35^ ^]^ Surprising, our results found that knockdown and overexpression of IGF2BP2 did not have an effect on CDK6 mRNA abundance, but instead produced the changes at the protein level. Huang et al. first proposed that the IGF2BP family can recognize and bind to m6A sites on mRNAs through its KH3‐4 structural domain and enhance mRNA stability and translation levels in an m6A‐dependent manner.^[^ ^18^ ^]^ Although they identified an active role of IGF2BP2 in the translation initiation phase, exactly how it regulates the translation process has not been explicitly investigated. Currently, different m6A readers use their own special structures or recruit different translation‐related proteins to play a variety of functions at different stages of translation.^[^ ^36^, ^37^ ^]^ YTHDC2 itself acts as an RNA helicase that unwinds the secondary structure of RNA and therefore plays an important role in translation elongation.^[^ ^11^ ^]^ HnRNPs are another m6A reader that uses its own structure to affect translation progression, and this class of proteins is able to regulate the cleavage form of precursor mRNA and thus affect the synthesis of subsequent proteins.^[^ ^38^, ^39^, ^40^ ^]^ Interestingly, METTL3, YTHDF1, and YTHDF3 are found to be able to promote mRNA translation progression through interaction with to EIF3, EIF4G, and other translation initiation factor proteins.^[^ ^19^, ^41^, ^42^, ^43^ ^]^ Therefore, we speculate that IGF2BP2 promotes translation output presumably by interacting with translation initiation factors, this also explains how it regulates the subsequent protein expression level of CDK6 without affecting the mRNA expression level.

IP‐MS identification revealed that EIF4A1 and EIF4A3 were potential interacting proteins of IGF2BP2. While the EIF4A, which consists of EIF4A1, 2, and 3, is an important component of the EIF4F initiation factor complex. EIF4A1 and 2 are highly similar in sequence and structure and play nearly similar roles in the translation process. In contrast, EIF4A3 is less similar to the preceding two and binds weakly to other key translation initiation factors such as EIF4G and EIF4E.^[^ ^44^ ^]^ It has been well documented that the EIF4A1 protein has an important role in the translation initiation process by recognizing and disassembling complex secondary structures in the 5′ ‐UTR, especially in the presence of abundant G‐quadruplexes, while facilitating the binding and scanning of ribosomes.^[^ ^45^, ^46^, ^47^ ^]^ We also tried to explore the binding of the CDK6 m6A modification site fragment RNA probe to the EIF4A1 protein by RNA pull‐down assays and found that they did not bind. Therefore, the binding site of EIF4A1 to CDK6 should be at the end of the 5′UTR region and not at the m6A modification site of CDK6. Our results revealed that the m6A modification sites on CDK6 mRNA were recognized and bound by IGF2BP2, which could perform protein interactions with EIF4A1 protein to facilitate the translation process by unraveling the complex secondary structure of the CDK6 mRNA initiation region. This provides powerful evidence that IGF2BP2 played an active role as an m6A reader in the translation initiation phase.

## 4 Conclusion

**Figure 8:**
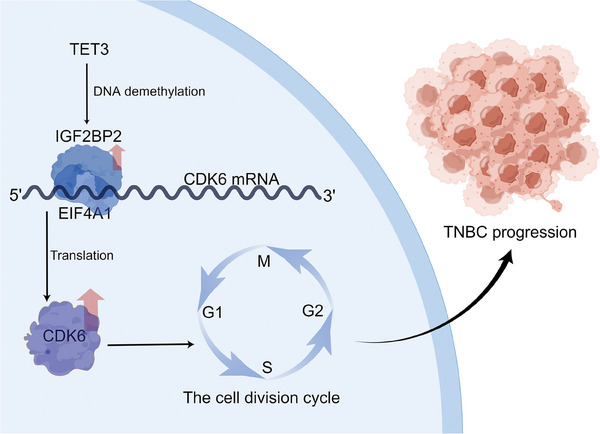
*Proposed model of IGF2BP2‐m6A‐CDK6 axis in TNBC*. IGF2BP2 was highly expressed in TNBC due to the lower methylation level in its promoter region by TET3. IGF2BP2 could recognize and bind to the m6A modification site located near the 5‐UTR of CDK6 mRNA and recruit EIF4A1 to facilitate the translation process of CDK6. And the elevated level of CDK6 protein further promotes the cell cycle progression, thus driving the proliferation of TNBC.

In this study, we found that IGF2BP2 was able to recruit the translation initiation factor EIF4A1 protein in TNBC, and exploited its key role in the cap structure‐dependent translation initiation process to increase the translation output of CDK6 in an m6A‐dependent manner. In turn, it driven the cell cycle from G1 to S phase and promoted TNBC cell proliferation (**Figure** **8**). In addition, knockdown of IGF2BP2 was able to increase the sensitivity of TNBC to abemaciclib, a CDK4/6 inhibitor. This would provide a potential therapeutic target for the diagnosis and treatment of TNBC, as well as a new strategy for broadening the drug indications for CDK4/6 inhibitors.

## 5 Experimental Section

### Public Databases Analysis

RNA‐seq and clinical data for targeted genes (IGF2BP1‐3, YTHDF1‐3, YTHDC1‐2) were obtained from the TCGA dataset (https://www.cancer.gov/about‐nci/organization/ccg/research/structural‐ genomics/tcga/using‐tcga/citing‐tcga), and heatmaps were drawn using Rstudio's complexheatmap package (https://github.com/jokergoo/ComplexHeatmap). Differential expression analysis of targeted genes (IGF2BP2, TET3) in breast cancer was obtained from bc‐GenExMiner 3.0 (http://bcgenex.ico.unicancer.fr/BC‐GEM). CpG islands were predicted and visualized from the Methprimer tool (http://www. urogene.org/methprimer/index.html). The prognostic value of IGF2BP2 in TNBC patients was analyzed using the Kaplan‐Meier mapper database (http://kmplot.com/analysis/). Expression analysis of CDK6 at the protein level in breast cancer solrile’ subtypes was obtained from the CPTAC database (http://ualcan.path.uab.edu/analysis‐prot.html). FIGDRAW (https://www.figdraw.com/static/index.html) was used to draw partial mechanism maps and schematic diagrams.

### Breast Cancer Tissue Samples

The TNBC tissues and adjacent normal tissues were obtained from Jiangsu Breast Disease Center, the First Affiliated Hospital with Nanjing Medical University. All patients' tissue samples were obtained before neoadjuvant chemotherapy or postoperative adjuvant chemotherapy and confirmed by histopathology. The above operations were reviewed and permitted by the Institutional Ethics Committee of the First Affiliated Hospital of Nanjing Medical University.

### Cell Lines and Cell Culture

The human breast cancer cell lines MCF‐7, ZR‐75‐1, SK‐BR‐3, BT‐474, MDA‐MB‐468, BT‐549, HCC‐1806, MDA‐MB‐231 were purchased from the American Strain Collection (ATCC, USA). The HCC‐1806 cell line was grown in RPMI 1640 (Wisent, China) culture, and the remaining cell lines were cultured in Dulbecco's modified eagle medium (DMEM) (Wisent, China) with 10% fetal bovine serum, 100 mg ml^−1^ streptomycin and 100 U ml^−1^ penicillin. All cells were cultured at 37 °C in a humidified incubator with 5% CO_2_.

### Lentivirus Transfection, Small Interfering RNA, and Plasmid

Lentiviral constructs were designed and synthesized by GenePharma (Shanghai, China) for the transfection of human breast cancer cells. Among them, it was referred to the LV3(H1/GFP&Puro) negative control vector as shRNA‐NC, and the cell lines that inhibit IGF2BP2 expression as shIGF2BP2‐1 and shIGF2BP2‐2. Similarly, it was referred to the LV5(EF‐1a/GFP&Puro) negative control Vector as vector and the cell line overexpressing IGF2BP2 as IGF2BP2. Puromycin (3 µg ml^−1^) was used for 2 weeks to screen stable transfected cell lines.

Small interfering RNAs (siRNAs) were designed and synthesized by GenePharma (Shanghai, China) to examine the effect of ten‐eleven translocation (TET) 1/2/3 and DNA‐methyltransferase (DNMT) 1/3A/3B on IGF2BP2 expression. Lipofectamine 3000 transfection reagent (Invitrogen, USA) was added to increase the transfection efficiency. The sequence of siRNAs was as follows: si‐TET1, 5′‐GCAAGACACCCAAGUCCUUTT‐3′, si‐TET2, 5′‐GGCAGUGCUAAUGCCUAAUTT‐3′, si‐TET3, 5′‐GGAAAUAAAGGCUGGUGAATT‐3′, si‐DNMT1, 5′‐GGAATGGCAGATGCCAACAGC‐3′, si‐DNMT3A, 5′‐CTACTACATCAGCAAGCGCAA‐3′, si‐DNMT3B, 5′‐AGATGACGGATGCCTAGAGTT‐3′.

The CDK6 overexpression plasmids were designed and synthesized by Corues Biotchnology (Nanjing, China) to carry out rescue experiment. The overexpression vector of CDK6 and its blank control were named as CDK6 and Vector. Lipofectamine 3000 transfection reagent (Invitrogen, USA) was added to increase transfection efficiency.

### RNA Extraction and Quantitative Real Time Polymerase Chain Reaction (qRT‐PCR)

Total RNA was extracted using Trizol reagent (TaKaRa, Japan) and ≈1000 ng RNA was reverse transcribed into complementary DNA (cDNA) using HiScript II Q RT SuperMix for qPCR (+gDNA wiper) (Vazyme, China). A real‐time PCR apparatus (Applied Biosystems, USA) was used to perform qRT‐PCR. Relative expression was calculated using the 2^−ΔΔCT^ or 2^−ΔCT^ method. PCR primers were designed as below, β‐actin forward, 5′‐ TCACCCACACTGTGCCCATCTACGA‐ 3′. β‐actin reverse, 5′‐ CAGCGGAACCGCTCATTGCCAATGG – 3′, IGF2BP2 forward, 5′‐CTACGCCTTCGTGGACTACC‐3′; IGF2BP2 reverse, 5′‐CATCCAACACCTCCCACTG‐3′; CDK6 forward, 5′‐CGTGGTCAGGTTGTTTGATG‐3′; CDK6 reverse, 5′‐CCTCGGAGAAGCTGAAACAT‐3′.

### Western Blot Analysis

Total proteins from human breast cancer cells were extracted using RIPA buffer (P0013C, Beyotime, China) containing 1% phosphatase inhibitor, 1% PMSF and 0.1% protease inhibitor. Total proteins were separated using 10% sodium dodecyl sulfate‐polyacrylamidegel electrophoresis (SDS‐PAGE) and transferred to PVDF (polyvinylidene fluoride) membranes (Millipore, USA). The membranes were placed in Tris‐buffered saline containing 0.1% Tween 20 (TBST) and 5% bovine serum albumin (BSA) for 2 h before the primary antibody was placed and left overnight at 4 °C. The primary antibody stock solution used was shown below. IGF2BP2 (1:1000, Abcam, UK, ab128175), GAPDH (1:1000, Beyotime, China, AF1186), CDK6 (1:1000, Protein‐tech, China, 14052‐1‐AP), EIF4A1 (1:500, Abcam, UK, ab31217), TET1 (1:1000, Affinity, USA, DF6428), TET2 (1:1000, Affinity, USA, DF12089), TET3 (1:1000, Affinity, USA, DF13335), CDK4 (1:1000, Protein‐tech, China, 11026‐1‐AP), CCND1 (1:1000, Protein‐tech, China, 26939‐1‐AP), pRb (1:500, Affinity, USA, AF0030). PVDF membranes were washed three times with TBST and incubated with secondary antibody (1:5000, Cell Signaling Technology, USA, 7074P2) for 2 h at room temperature. Immobilob Western Chemiluminescent HRP Substrate (Millipore, USA) was used to detect the expression level of the target protein.

### Detection of Methylation Levels

To detect the methylation level of IGF2BP2 promoter region, DNA was extracted from human breast cancer cells using a DNA extraction kit (TIANamp Genomic DNA Kit, TIANGEN, China), and then DNA Bisulffte Conversion Kit (TIANGEN, China) was used for DNA bisulfate conversion. Primers were designed using the Methprimer tool (http://www.urogene.org/methprimer/index.html) and amplified using a real‐time PCR instrument. Finally, PCR amplification products were further analyzed by agarose gel electrophoresis. The primer sequences were shown as below. Methylation forward, 5′‐GTTATTTTAGAGAGCGTGGCG‐3′, Methylation reverse, 5′‐AAATACGCTACTACCAAAACAAACG‐3′, Unmethylated forward, 5′‐TATTTTAGAGAGTGTGGTGGGG‐3′, Unmethylated reverse, 5′‐ACACTACTACCAAAACAAACAAAA‐3′.

### CCK‐8 Assay

The proliferation of human breast cancer cells was examined according to the instructions of CCK‐8 kit (Vazyme, China). Two thousand cells were evenly grown in 96‐well plates and cultured with DMEM/RPMI1640 medium containing 10% fetal bovine serum for 5 days. After adding CCK8 reagent at the same time point each day, the cells were incubated in a cell incubator for 2.5 h protected from light, and the absorbance was measured at 450 nm using an enzyme marker (Groding, Tecan, Austria) and recorded.

### Colony Formation Assay

Five hundred individual breast cancer cells were evenly grown in 6‐well plates and incubated in a cell culture incubator for 2 weeks. Colonies were fixed with paraformaldehyde and stained with crystal violet stain (Beyotime, China). After drying, the colonies were photographed and counted using Image J and recorded.

### 5‐Ethynyl‐2′‐Deoxyuridine (EdU) Assay

Twenty thousand individual breast cancer cells were uniformly grown in 96‐well plates and placed in the incubator for 24 h. The next day, the cells were incubated and fixed using EdU, 4% paraformaldehyde, and 0.5% Triton X‐100 according to the instructions provided with the BryoCliclick EdU‐555 Cell Proliferation Assay Kit (Beyotime, China). Finally, staining was performed using the configured Click Additive Solution and Hoechst 33342 and observed and photographed under a fluorescence microscope (Nikon, Japan).

### Cell Cycle Assay

Experiments were performed according to the instructions as provided in the cell cycle staining kit (MultiSciences Biotech, China, CCS012). Briefly, one million breast cancer cells were collected and washed twice with phosphate buffered saline (PBS) and centrifuged (1000 rpm, 5 min). Cells were then resuspended with 1 ml of DNA staining solution and 10 µl of Permeabilization solution and incubated for 30 min at room temperature and protected from light. Data were analyzed using FlowJo v10.0 (FlowJo, LLC).

### Xenograft Tumors in Nude Mice

The animals and operations involved in this experiment were approved by the Animal Ethics Committee of Nanjing Medical University (IACUC‐2206017), and the experiments were performed in strict accordance with the official documents. In the first experiment, female BALB/c nude mice (4‐6 weeks old, 18–22 g) were randomly divided into 2 groups of 6 mice each, named shRNA‐NC and shIGF2BP2‐1. In the second experiment, nude mice were randomly divided into 4 groups of 6 mice each, named shRNA‐NC+Vector, shRNA‐NC+CDK6, shIGF2BP2+Vector, shIGF2BP2‐1+CDK6. Each mouse was inoculated with one million MDA‐MB‐231 cells subcutaneously in the right abdomen of nude mice. Subcutaneous tumor volumes were measured every 4 days. The mice were euthanized after 4 weeks and final tumor weights were recorded.

### Protein Stability Assay

Cycloheximide (CHX, HY‐12320; MCE, USA) was dissolved in DMSO and rationed to 20 µg/ml. Human breast cancer cells were placed uniformly in a six‐well plate and the above solution was added at 0 h, 1 h, 2 h, 4 h, 6 h, and 8 h and the protein levels of CDK6 were detected according to the above Western blot procedure.

### RNA Immunoprecipitation (RIP) Assay and RIP‐Seq

RIP assays were performed according to the experimental procedures provided in the Magna RIP Kit (Millipore, USA). Cell lysates were prepared using RIP lysate and then incubated with 5 mg of antibody at 4 °C overnight. The following day RNA‐protein immunocomplexes were collected using Protein A/G magnetic beads. The purified RNA was analyzed by RT‐PCR and qRT‐PCR, and the amplified products were further analyzed and displayed using agarose gel electrophoresis. Three independent replicate experiments were performed using MDA‐MB‐231 wild‐type cells, and the purified RIP products were sent to RiboBio Co., Ltd. (Guangzhou, China) for library building and high‐throughput sequencing.

In addition, total RNAs of shRNA‐NC and shIGF2BP2‐1 were extracted from three sets of MDA‐MB‐231 cells and sent them to RiboBio Co., Ltd. (Guangzhou, China) for mRNA‐sequencing.

### Dual‐Luciferase Reporter Assay

Dual luciferase plasmids were synthesized by Corues Biotchnology (Nanjing, China) and used to identify potential m6A recognition sites. Human breast cancer cells were uniformly grown in 6‐well plates and transfected with Lipofectamine 3000 transfection reagent (Invitrogen, USA) and left in the cell incubator for 2 days. On the day of the assay, cells were sufficiently lysed using reporter gene lysis solution and centrifuged (15 000 g for 3 min), and the supernatant was used for the assay. The luciferase activity was measured and the results were analyzed strictly according to the instructions provided in the Dual Luciferase Reporter Gene Assay Kit (Beyotime, China).

### Methylated RNA Immunoprecipitation (MeRIP) Assay and MeRIP‐Seq

MeRIP assays were performed according to the experimental procedures provided in the Magna MeRIP m6A Kit (Millipore, USA). Total RNA was extracted using Trizol reagent (TaKaRa, Japan) and fragmented into fragments of about 100 nt length. The fragmented RNA obtained was incubated with m6A antibody (Anti‐N6‐methyladenosine, abcam, UK, ab208577) or murine IgG. Immunoprecipitated complexes were collected using Protein A/G magnetic beads and purified and preserved. Samples from three independent replicate experiments using MDA‐MB‐231 cells were sent to Ovation Genetics (Beijing, China) for library construction and high‐throughput sequencing. In addition, it was designed specific primers for qRT‐PCR to detect the expression of m6A‐modified genes, and the sequences were shown as below. CDK6‐A‐forward, 5′‐CTGCAGGGAAAGAAAAGTGC‐3′, CDK6‐A‐reverse, 5′‐CCTCGAAGCGAAGTCCTCAA‐3′, CDK6‐B1‐forward, 5′‐CAAGACTTGACCACTTACTTGGA‐3′, CDK6‐B1‐reverse, 5′‐GAAAGTCCAGACCTCGGAGA‐3′, CDK6‐B2‐forward, 5′‐GTGACCAGCAGCGGACAAA‐3′, CDK6‐B2‐reverse, 5′‐ACTGAGGTTAGAGCCATCTGGAA‐3′.

### Co‐Immunoprecipitation (COIP) Assay and Immunoprecipitation and Mass Spectrometry (IP‐MS)

The COIP assay was performed basically according to the instructions provided by MedChemExpress (USA). Briefly, human breast cancer cells were washed twice with ice‐cold PBS and fully lysed with 200 µl of lysis buffer (50 mM Tris‐HCl, pH 7.5, 150 mM NaCl, 15 mM MgCl_2_, 5 mM EDTA, and 0.1% NP‐40) containing Protease inhibitor cocktail (Beyotime, China, P1008). Protein A/G magnetic beads (MCE, USA, HY‐K0202) were washed and mixed with antibodies by rotation at 4 °C for 4 h. Magbeads‐Ab complexes were mixed thoroughly with cell lysates at 4 °C and left overnight. Immunoprecipitates were collected and used for Western blot assay after 3 times of thorough washing with IP lysate. Three independent replicate experiments were also performed and sent the samples to Beijing Genomics Institute (China) for mass spectrometry analysis to obtain potential interacting proteins of IGF2BP2.

### RNA‐Pull Down Assay

Streptavidin magnetic beads (MCE, USA, HY‐K0208) were used for the RNA pull‐down assay, and biotinylated RNA was designed and synthesized by Shanghai Generay Biotech Co. Human breast cancer cells were washed three times with ice‐cold PBS and cell lysis reagent (using a lysate formulation above in Western blot assay) containing RNase Inhibitor (Beyotime, R0102, China) was added to obtain protein lysates. The biotinylated probe, protein lysate and streptavidin magnetic beads were mixed and incubated. Ultimately the complexes were boiled and used for Western blot assay.

### Statistical Analysis

Unless otherwise stated, all experiments were performed in principle with at least three independent replications. Data were calculated and organized and plotted using GraphPad Prism (Version 8.0). Student's t test was used to test whether the differences between study groups were statistically significant, and p < 0.05 was used as the criterion for statistical significance.

## Conflict of Interest

The authors declare no conflict of interest.

## Author Contributions

T.X., X.Y‐D., and M.Y.‐S. contributed equally to this work. Q.D. and J.F.‐W. conceived and designed the project. T.X., X.Y‐D., and M.Y.‐S. performed the experiments and conducted the data analysis. X.Z., F.X., J.W., and L.S. helped with experiments and provided valuable advice. T.X. and J.F.‐W. wrote and revised the manuscript. All authors read and approved the final manuscript.

## Supporting information

Supporting Information

## Data Availability

The data that support the findings of this study are available from the corresponding author upon reasonable request.
