# Supplementary material for: IGF2BP2 Drives Cell Cycle Progression in Triple‐Negative Breast Cancer by Recruiting EIF4A1 to Promote the m6A‐Modified CDK6 Translation Initiation Process
Source: Adv Sci (Weinh). 2023 Nov 20;11(1):2305142. doi: 10.1002/advs.202305142 (PMC10767445; doi:10.1002/advs.202305142)
Supplement: Supplementary file 1 — Supporting Information [file ADVS-11-2305142-s001.pdf]

ADVANCED SCIENCE
Open Access

Supporting Information

for *Adv. Sci.*, DOI 10.1002/advs.202305142

IGF2BP2 Drives Cell Cycle Progression in Triple-Negative Breast Cancer by Recruiting EIF4A1 to Promote the m6A-Modified CDK6 Translation Initiation Process

Tian Xia, Xin-Yuan Dai, Ming-Yi Sang, Xu Zhang, Feng Xu, Jing Wu, Liang Shi, Ji-Fu Wei and Qiang Ding**

A

Variables	Total patients	Low expression of IGF2BP2	High expression of IGF2BP2	p Value Chi-squared test
All cases	40	11	29	
Age				
≤50	14	5	9	
>50	26	6	20	0.3932
TNM stage				
I-II	37	11	26	
III	3	0	3	0.2674
Tumor size				
≤2 cm	12	7	5	
>2 cm	28	4	24	0.0042**
Metastatic axillary lymph nodes				
0	24	8	16	
≥1	16	3	13	0.3116
Histological grade				
I-II	6	3	3	
III	34	8	26	0.1806

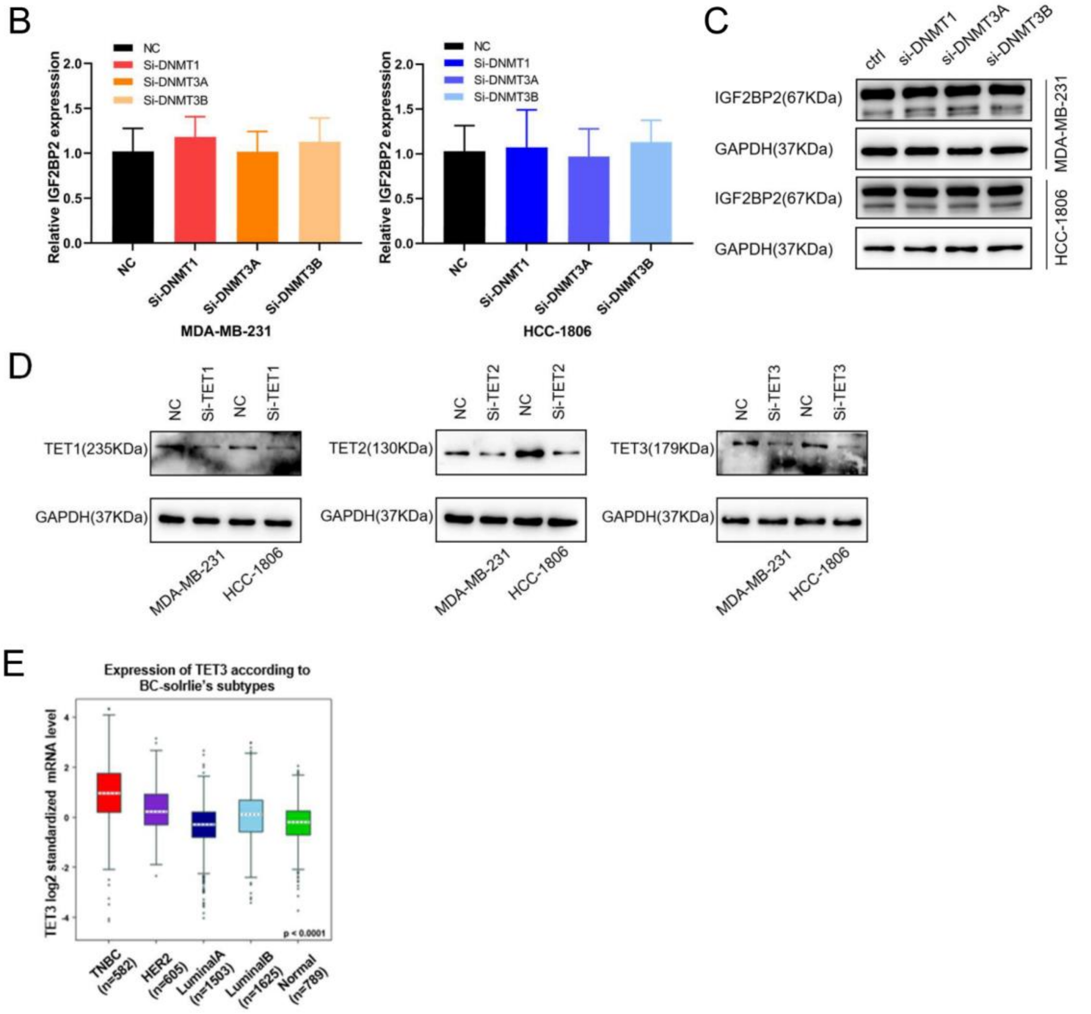

Supplementary Figure S1

(**A**) Correlation between IGF2BP2 expression and clinicopathological characteristics of 40 TNBC patients. (**B and C**) QRT-PCR (B) and western blot (C) analysis of IGF2BP2 mRNA expression in MDA-MB-231 and HCC-1806

cells transfected with si-DNMT1/3A/3B. (**D**) TET1/2/3 expression in MDA-MB-231 and HCC-1806 cell lines transfected with si-TET1/2/3 was analyzed through Western blot assays. **(E)** Expression of TET3 in solrile's subtypes of breast cancer analyzed by bc-GenExMine tool. Data were shown as mean ± SD. *p <0.05, **p <0.01, ***p<0.001.

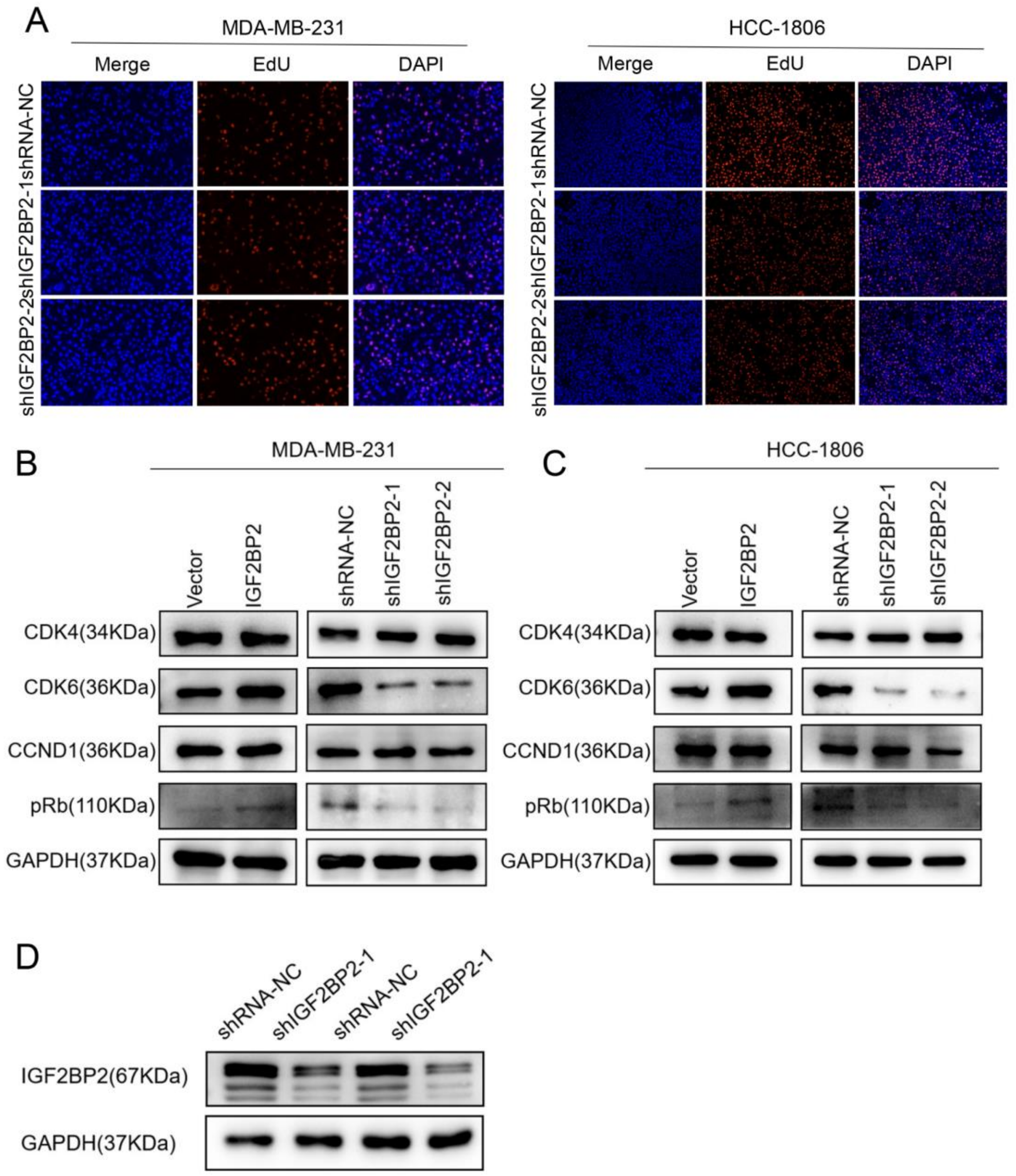

Supplementary Figure S2

(**A**) EdU assays was used to measure the proliferation of cells in IGF2BP2-

knockdown and control group, and representative pictures were shown above. (**B** and **C**) Western blot analysis of protein expression of cell cycle G1/S phase-related proteins in lentiviral stabilized cell lines of IGF2BP2. **(D)** Western blot analysis of IGF2BP2 expression in different groups of tumors.

A

All 13 targets genes enriched in cell division(GO:0051301)

Location	Peak start	Peak end	Gene ID	Symbol	Description
chr14	99510200	99510400	8812	CCNK	Cyclin K
chr7	92606800	92607000	1021	CDK6	Cyclin dependent kinase 6
chr16	89645800	89646200	5119	CHMP1A	Charged multivesicular body protein 1A
chr17	59619800	59620000	1213	CLTC	Clathrin heavy chain
chr14	101964600	101964800	1778	DYNC1H1	Dynein cytoplasmic 1 heavy chain 1
chr1	108877000	108877200	29899	GPSM2	G protein signaling modulator 2
chr1	244857600	244857800	3192	HNRNPU	Heterogeneous nuclear ribonucleoprotein U
chr10	92649800	92650000	3832	KIF11	Kinesin family member 11
chr7	40134200	40134600	136647	MPLKIP	M-phase specific PLK1 interacting protein
chr4	39848800	39849000	23244	PDS5A	PDS5 cohesin associated factor A
chr1	151405200	151405400	23126	POGZ	Pogo transposable element derived with ZNF domain
chr9	104125000	104125200	10592	SMC2	Structural maintenance of chromosomes 2
chr10	86453200	86453400	23063	WAPL	WAPL cohesin release factor

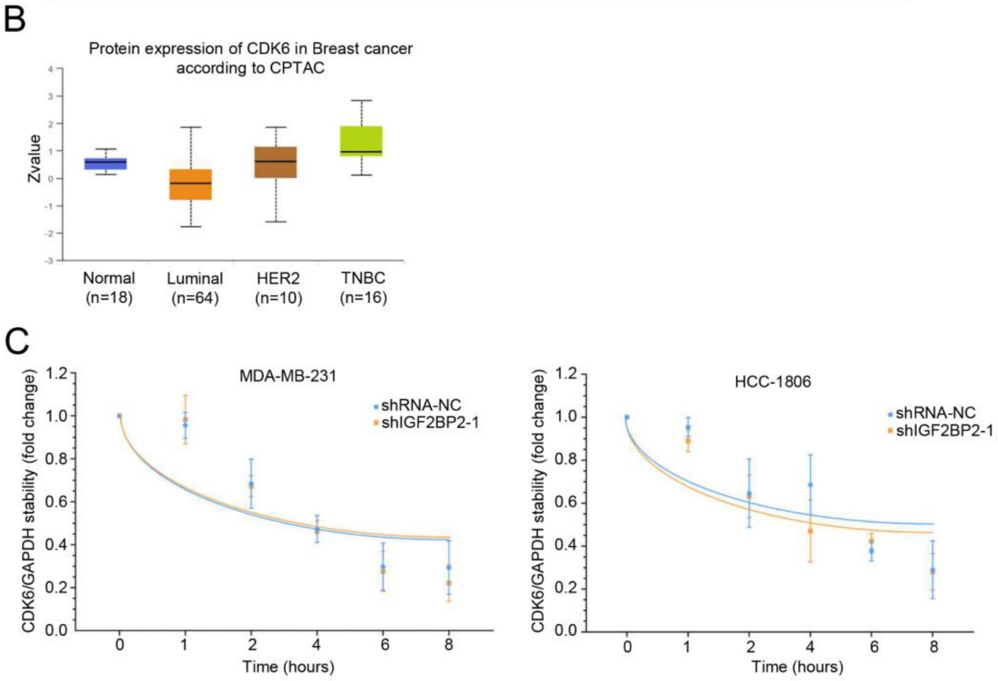

Supplementary Figure S3

(**A**) The list of potential IGF2BP2 target genes in cell division (GO: 0051301). (**B**) The protein levels of CDK6 in different subtypes of breast cancer based on CPTAC database. (**C**) ImageJ was used to analyze the gray value of the CHX

assays results, and the numerical were displayed by curves.

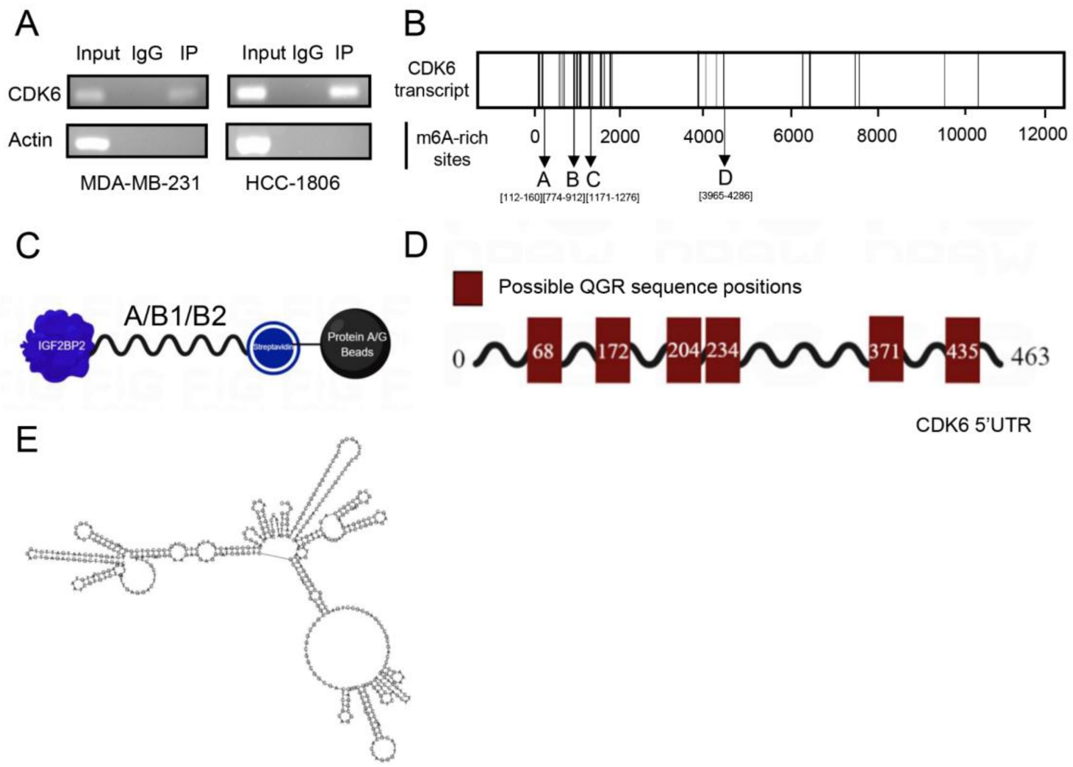

Supplementary Figure S4

(**A**) Agarose gel electrophoresis showed the results of RIP-qRT-PCR. (**B**) Scramp tool (http://www.cuilab.cn/sramp) was used to predict the possible m6A modification sites on CDK6 mRNA. (**C**) Model of biotinylated CDK6 probe. (**D**) QGRS Mapper (https:// bioinformatics.ramapo.edu/QGRS/index.php) was used to predict the G quadruplex structure. (**E**) RNA fold web server (http://rna.tbi.univie.ac.at/cgi-bin/RNAWebSuite) was used to analyze the secondary structure of CDK6 5’UTR region.

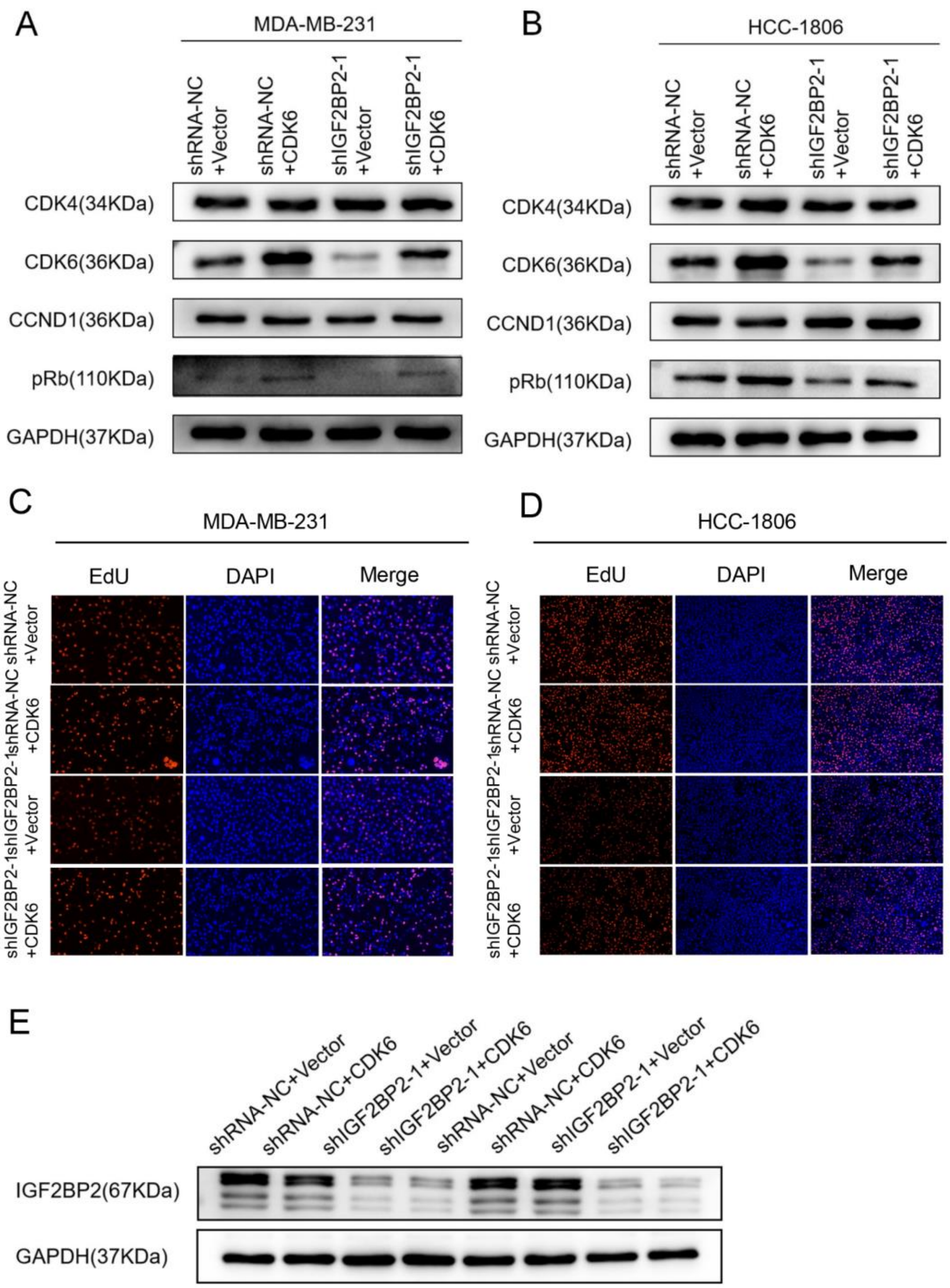

Supplementary Figure S5

(**A**-**B**) Western blot analysis of cell cycle G1/S phase-associated protein expression after overexpression of CDK6 in IGF2BP2 lentiviral stable cell line. (**C**-**D**) EdU assays was used to measure the proliferation of cells in each group of reversion experiments, and representative pictures were shown above. (**E**) Western blot analysis of IGF2BP2 expression in different groups of tumors in

the rescue experiment.

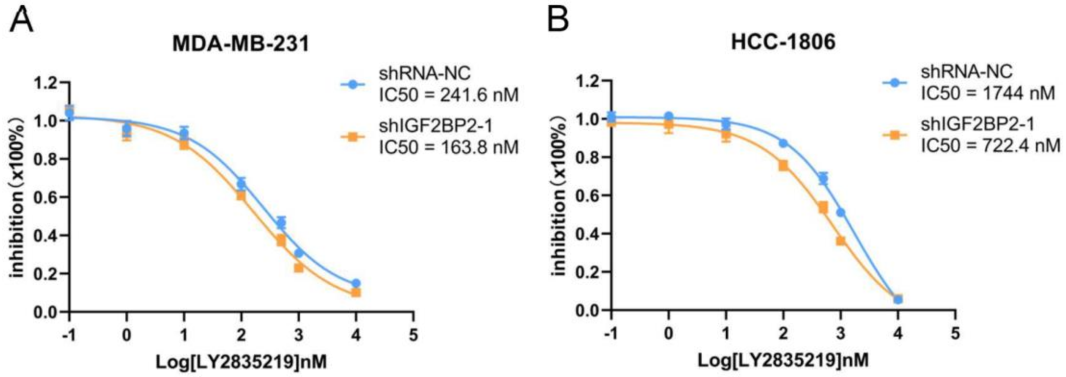

Supplementary Figure S6

(**A**-**B**) CCK-8 assay was used to examine the IC50 value of abemaciclib (Abe, LY2835219).
